# Species Identification in Drosophilids; the Utility of k‐mer Characters

**DOI:** 10.1111/mec.70624

**Published:** 2026-11-06

**Authors:** Rob DeSalle

**Affiliations:** ^1^ American Museum of Natural History New York New York USA

## Abstract

Over 14,900 COI DNA sequences of drosophilids were used to build a character‐based diagnostic system for the identification of genera and species in the group. This approach uses aligned COI sequence characters, k‐mer characters and applies a hybrid approach that combines the two. To assess the efficacy of the diagnostics, accuracy versus coverage plots were used and resulted in the observation that practical rules (allowing 5% leakage [misidentified assignments]) perform much better than strict rules (no leakage). In addition, *k* = 10 was determined as the optimal *k* value for k‐mer character analysis. Hybrid methods incorporating both k‐mer characters and aligned DNA sequence characters outperform the individual approaches. Finally, unnamed drosophilid accessions were compiled and tentatively assigned taxonomy based on the diagnostic system developed here.

## 1 Introduction

*Drosophila melanogaster* and its close relatives have long been an evolutionary paradigm. While this fly centric paradigm is over a century old, expansion of interest away from the model organism *D. melanogaster* has exploded in the last few decades (Suvorov et al. 2022; Kim et al. 2024; Kim et al. 2021; O'Grady and DeSalle 2018; Li et al. 2022; DeSalle et al. 2022; Finet et al. 2021). This interest in the family Drosophilidae has also been accompanied by a renewal of interest in taxonomy and field work involving the group. Identification of the flies in this group using morphology is advanced (several keys exist to facilitate identification (McAlpine et al. 1981; Markow and O'Grady 2005; Buck et al. 2009; Marshall et al. 2016; Borkent and Rotheray 2009; Miller et al. 2017; Yuzuki and Tidon 2020; Grimaldi 1990)) and allows for accurate identification of specimens.

DNA sequence identification in the guise of DNA barcoding (Hebert et al. 2003; Fišer Pečnikar and Buzan 2014; Hebert and Gregory 2005; DeSalle et al. 2005; Robert DeSalle 2024) has also been applied to some of the taxa in Diptera (Yassin et al. 2010; Yassin et al. 2008; Meier et al. 2006; Fu et al. 2016; Zhang and Zhang 2014; Mitra et al. 2025). While Meier et al. (2006) questioned the utility of DNA barcoding in Diptera in general, Yassin et al. (2010) demonstrated that character‐based barcoding in the Drosophilidae was a valid approach to species identification in the family. Distance approaches with a barcode gap are by far the preferred method for barcode interpretation, but some researchers have expressed interest in character‐based approaches as well (DeSalle and Goldstein 2019; Mitra et al. 2025; Goldstein and DeSalle 2011; Rach et al. 2008; Weitschek et al. 2014; Bertolazzi et al. 2009).

In this paper a DNA diagnostic system for identification of genera and species in the family Drosophilidae is developed using a character‐based approach. Aligned DNA sequences (ALIGN) and k‐mers (KMER) were used as a source of characters for the diagnostic systems. The dynamics and behaviour of both single column and paired column diagnostics and their appropriateness for species identification were examined. Short COI sequences, the standard DNA barcode for animals (Hebert et al. 2003), were strictly used.

## 2 Materials and Methods

### 2.1 Dataset

Drosophilidae COI sequences in the NCBI database were downloaded. The download totalled 17,488 when the query “Drosophilidae + COI” was used. The first filter was to remove all non drosophilid or non‐closely related dipterans from the dataset which resulted in 16,777 sequences. The resulting sequence (COI_UNALIGNED_RAW.fasta in supplemental data 1 on DRYAD) accessions were then filtered to include only sequences of greater than 300 bases. This resulted in a list of 14,900 sequences (COI_UNALIGNED_FINAL.fasta and COI_ALIGNED_FINAL.fasta in supplemental data 1 on DRYAD). The taxonomic distribution was widespread across the family (see truth table in supplemental table 1 in DRYAD) and also included other closely related dipteran taxa (Ephydridae and Stegana). Each accession number was “truthed” with regard to species and genus name taken from the NCBI taxonomy. Some accessions had no taxonomy (415 sequences; NA NA called NANA), and some had genus only taxonomy (2315 sequences; e.g., *Drosophila* NA called GENA). The truth files are used in rule building and in identifying sequences during rule testing.

### 2.2 Alignment and Data Disposition

Greater than 14,900 COI sequences were aligned using MAFFT (setting = mafft ‐‐add QUERY.fasta \ ‐‐keeplength \BACKBONE_ALIGNMENT.fasta \ > PROJECTED_ALIGNMENT.fasta). Because avoidance of gaps in the diagnostic approach was desired, ends of the alignment were trimmed to leave only columns with 95% or greater occupation. The alignment was then masked to have only columns that are in the *D. melanogaster* reference. This left us with 614 high quality aligned columns with coordinates established on the reference *D. melanogaster* COI sequence running from base 183 from the 5′ end of the reference COI gene to position 598 bp from the 5′ end of the reference gene. This study examines the utility of both aligned DNA (ALIGN) sequences using columns of the alignment as characters and k‐mer (KMER) based characters independent of alignment. The aligned sequences described above were used for all aligned/masked/DNA sequence analyses (ALIGN) and the raw fasta sequences were used for the k‐mer analyses (KMER).

### 2.3 Splitting the Data Set

In order to provide a test data set for evaluating the rules that are built, the 14,900 fasta sequences were split into a training set (50% of the accessions) and a test data set (the other 50% of the accessions). Analyses using other taxonomic systems (Data not shown) indicate that all splits (30/70, 40/60, 50/50, 60/40 and 70/30) give similar and reasonable results, so maximal training set size (50%) with maximal test set size (50%) was selected. The splits were accomplished in a random but stratified manner (at least two sequences for each taxon had to be in both the train and test datasets).

### 2.4 Build Diagnostic Rules

All manipulation of DNA sequences and k‐mer analysis (KMER) were accomplished using a library of custom scripts (see DRYAD; https://doi.org/10.5061/dryad.ghx3ffc4k). The approach to building characters for ALIGN COI sequences allows us to retrieve five categories of rules (see Figure **1**)—S, SP, P, PP and C; these are roughly ordered from low complexity to higher complexity. Figure 1 demonstrates how these characters are obtained. S and SP are single column rules that can diagnose a taxon and P and PP are paired columns that can be built for clear diagnosis of taxa in the dataset. The C category is simply S + SP + P + PP.

**FIGURE 1:**
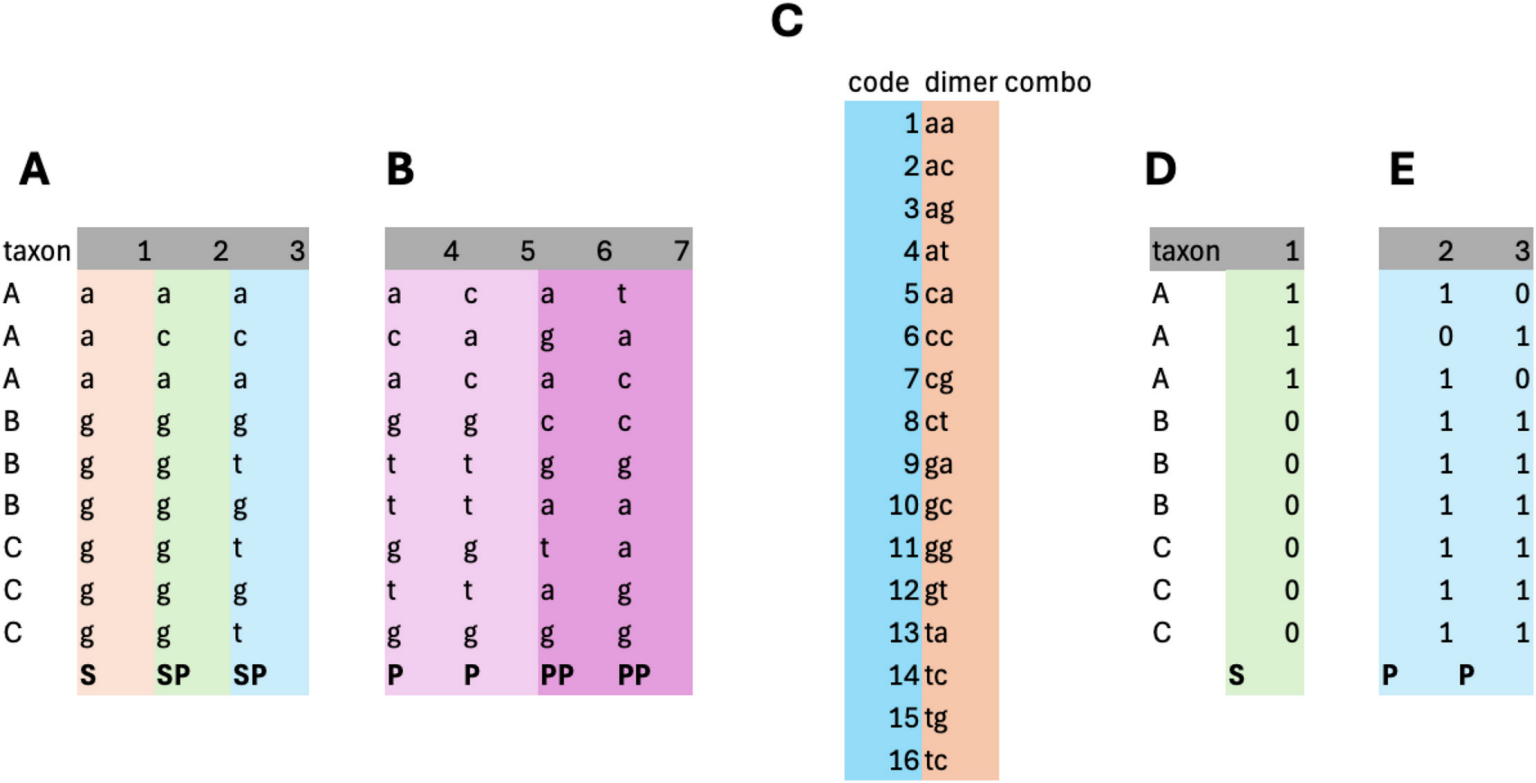
Single and paired column diagnostics (A, B) and their reduction in k‐mer space (C–E). Sequences from nine individuals representing three taxa (A–C), with three individuals per taxon, are shown to illustrate diagnostic character classes. (A) Single‐column diagnostics. Position 1 represents the simplest diagnostic case (S), in which a single nucleotide state uniquely identifies a taxon. Positions 2 and 3 illustrate more complex single‐column pattern diagnostics (SP), where multiple states collectively diagnose the taxon relative to outgroups. (B) Paired‐column diagnostics. Columns 4–7 show combinations of two alignment positions. Columns 4 and 5 illustrate simple paired diagnostics (P), where a small set of nucleotide combinations uniquely distinguishes a taxon. Columns 6 and 7 illustrate paired pattern diagnostics (PP), in which larger, non‐overlapping sets of combinations define the taxon. (C) Enumeration of the 16 possible nucleotide pair combinations (dimers), used to formalize paired‐column diagnostics. (D, E) Reduction to presence/absence character space. When sequence data are converted to binary presence/absence representations (e.g., k‐mer matrices), only two character states are available. As a result, pattern‐based categories (SP and PP) collapse, leaving only single (S) and paired (P) diagnostic classes.

k‐mers are an independent method of obtaining information from DNA sequences. One can use k‐mer information to compute distances or as done in this paper as characters. k‐mer‐based diagnostics were constructed using binary presence/absence representations of genomes. For each value of *k* (*k* = 9–14) an initial library of COI k‐mers is constructed and each of the 14,900 COI sequences is scored for presence/absence of each individual k‐mer. This method results in a presence absence matrix with 14,900 taxa and over 50,000 k‐mer columns. Because k‐mer data constructed in this way are binary (0,1), the diagnostic hierarchy is reduced to three rule classes: S (single k‐mers), P (k‐mer combinations) and C (combined rules), as SP and PP cannot be distinguished in a two‐state system (Figure 1).

The rule build step for paired or combinatorial rules is both computationally intense and sporadic. If too many paired columns are examined for rule extraction, computational time increases exponentially. If too few paired columns are examined, only small numbers of combinatorial rules are recovered. The number of paired columns was optimized with rule building and computational time by running a systematic sweep of paired column numbers (*n* = 20,000, 200,000, 500,000, 1,000,000 and 2,000,000).

Rules for both ALIGN sequences and KMER analysis are then built the same way. Rules are constructed under STRICT (100% of the target taxa are the same state) and PRACTICAL (95% of the target taxon are the same state) criteria. Because the sequence of the COI gene is only 600 bases, this limits the number of paired columns that need to be examined and allows us to exhaustively search all paired columns possible with reasonable computation time. In addition, separate rule sets are built for the two taxonomic levels (genus and species) examined in this paper.

### 2.5 k‐mer Sweeps

To identify the optimal k‐mer length, a k‐mer sweep was performed across *k* = 9–15. Optimal *k* length in phylogenetic studies have been suggested to be in this range (Hall and Nisbet 2023; Bussi et al. 2021; Zhang et al. 2017). For each *k*, rules were derived from the training dataset and evaluated on the left‐over independent test dataset. Performance was assessed using accuracy–coverage profiles and ROC (Receiver Operating Characteristic; Gonçalves et al. 2014) curves, including AUC (Area Under the Curve) for individual rule classes and combined classifiers. The optimal *k* was selected based on high AUC, consistent performance across taxonomic levels and stability across evaluation datasets. To evaluate whether combining aligned nucleotide features (character states) and k‐mer features improves taxonomic diagnosability, a hybrid diagnostic framework integrating both data types within a shared feature space was built.

### 2.6 Matrices

All analyses were performed under a strict train–test separation, and no features were derived independently from the test set. k‐mer presence/absence matrices were generated using *k* = 10, which was previously identified as optimal. A shared k‐mer index was constructed from the training set and subsequently applied to the test set to ensure consistent feature representation. Hybrid feature matrices were constructed by concatenating: Aligned nucleotide positions, encoded as one‐hot variables (A/C/G/T per site) and k‐mer presence/absence features.

### 2.7 Rule Construction and Hybrid Diagnostics

For each taxon, candidate diagnostic features were evaluated using the difference between within‐taxon and outside‐taxon frequencies (score = in_frac − out_frac). Features were ranked by this score, and the top T features per taxon (T = top features) were retained (TOP_T ∈ {20, 30, 50}). Only features with positive discriminative value were considered. Taxonomic assignment was performed using weighted voting, where each feature contributed a vote proportional to its discriminative score. To reduce bias toward taxa with larger rule sets, votes were normalized per taxon. Vote normalization rescales the cumulative diagnostic votes for each candidate taxon so that taxa with many diagnostic features do not receive an inflated value simply because they possess larger rule sets. A margin‐based acceptance criterion (or threshold) was applied (i.e., assignment accepted only if (best_score − second_best_score) ≥ a preset margin). Margin values ranging from 0.0 to 0.5 were explored.

### 2.8 Optimization

Model performance was evaluated across combinations of (1) TOP_T ∈ {20, 30, 50}, (2) normalization ∈ {True, False} and (3) margin ∈ {0.0, 0.1, 0.2, 0.5}. Performance metrics included: (1) Coverage (COV): proportion of sequences assigned, (2) Accuracy (ACC): proportion of correct assignments among assigned sequences, (3) Exact match (EXACT) and (4) Average set size (AVG_SET). The optimal configuration was selected based on maximizing accuracy while maintaining substantial coverage.

### 2.9 Aggregate Diagnostic Analysis

To evaluate hierarchical recovery of taxonomic signal, aggregate diagnostic analyses were performed by combining diagnostic rules derived at multiple taxonomic levels into a unified assignment framework. For aggregate genus analysis, both genus‐level rules and species‐level rules collapsed to genus identity were pooled into a single diagnostic space. Species‐derived rules contributed support to their corresponding genus, allowing recovery of higher‐level signal from lower‐level diagnostic structure. Aggregate analyses were performed independently for ALIGN, KMER and HYBRID systems under both strict and practical rule‐generation regimes (regime is simply whether practical or strict rules were used).

### 2.10 Hierarchical Assignment: Taxonomic Rescue

To examine how diagnostic signal behaves across taxonomic levels and sequence representations, assignment outputs from alignment‐derived positional DNA (ALIGN), k‐mer presence/absence (KMER) and hybrid integrated (HYBRID) classifiers were transformed so that comparison of the dynamics of accuracy and coverage across rule sets could be accomplished. Two inference regimes were evaluated. In the NANA regime, query sequences were treated as taxonomically unconstrained, with both genus and species unknown. In the GENA regime, genus identity was treated as known while species identity remained unknown, thereby restricting competition to species within a fixed genus‐level context. For each query accession, assignment confidence was quantified as the difference between the highest and second‐highest assignment scores (score1 − score2), whereas assignment exclusivity was quantified as the logarithm of the ratio between the top two scores (log10(score1/score2)). Species‐level universes were constructed directly from species assignment scores. Genus‐level assignments were constructed by collapsing full species score distributions into genus‐level aggregates by summing scores across all species belonging to each genus prior to ranking. Heatmaps were generated for each representation and inference regime using identical row ordering based on decreasing species‐level confidence, allowing direct comparison of hierarchical stabilization and ambiguity structure across diagnostic representations. Hybrid aggregate analyses combined positional ALIGN diagnostics and k‐mer diagnostics within a shared scoring framework to evaluate complementary recovery of taxonomic signal across methods.

### 2.11 Scripts and Datafiles

All scripts are available at DRYAD (https://doi.org/10.5061/dryad.ghx3ffc4k). Scripts are in Scripts.zip and data are in Data_files.zip. The relevant files on Dryad are called Data_files.zip.

### 2.12 AI Statement

The scripts in this publication were generated with the assistance of CHATGPT. They were also rigorously tested and refined by the author. Some of the figures were also generated with the assistance of AI scripting and then edited if needed.

## 3 Results

### 3.1 Rule Counts

The algorithm for generating rules is computationally greedy, especially with regard to the building of paired diagnostics. Hence, the lower limit of the number of paired columns needed in the rule build phase of the approach was determined.

Figure 2 demonstrates that at low exploration numbers (20,000 and 200,000) column pairs diagnostic structure is dominated by singleton rules, particularly at the species level where P/S ratios remain near zero. Increasing exploration depth (1,000,000 and 2,000,000) results in rapid nonlinear recovery of pairwise rules, with pairwise signal eventually exceeding singleton signal under both genus and species strict regimes. Species‐level practical and strict regimes exhibit especially strong combinatorial expansion, demonstrating that substantial diagnostic information is embedded in higher‐order compositional structure that remains hidden under sparse pair sampling. Figure 2 also demonstrates a steady increase in paired column rule candidates. The largest jump in the sweep sits between 1 million and 2 million paired columns (Figure 2). Larger n was not examined but based on computational time it was determined that 2 million paired columns would be the best tradeoff. More columns will obviously increase the number of paired column rules, but it will also cause runtime to expand exponentially.

**FIGURE 2:**
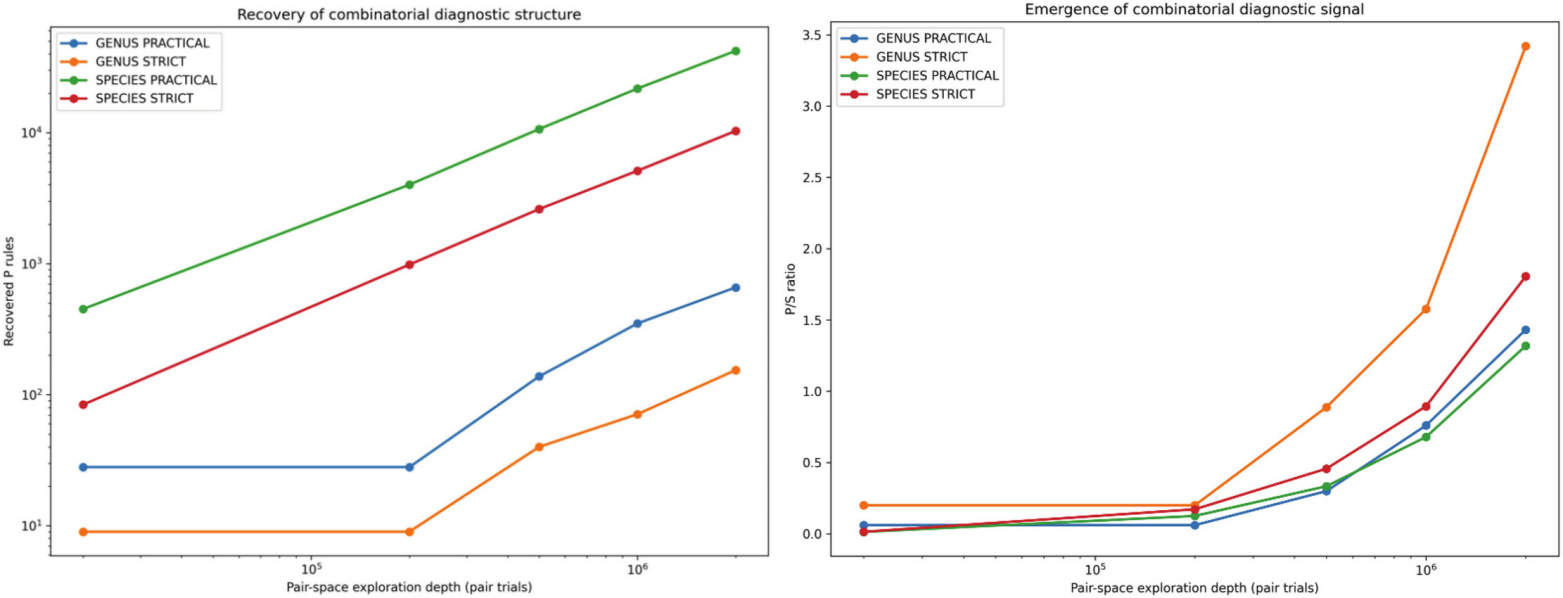
Emergence and recovery of combinatorial diagnostic signal as a function of pair‐space exploration. Pairwise diagnostic (P) rules were reconstructed from *k* = 10 presence/absence matrices across increasing pair‐space exploration depths (20k–2M sampled pair combinations) under genus and species taxonomic levels using strict and practical rule regimes. Left panel shows the total number of recovered P rules on a logarithmic scale. Right panel shows the ratio of pairwise to singleton rules (P/S), reflecting the relative contribution of combinatorial versus singleton diagnostic signal.

The dynamics of rule building using the aligned masked DNA sequence data set and the KMER data set were next examined (Figure 3). Figure 3A (Supplemental Table S2) shows the distribution of character categories obtained from the ALIGN sequence data set.

**FIGURE 3:**
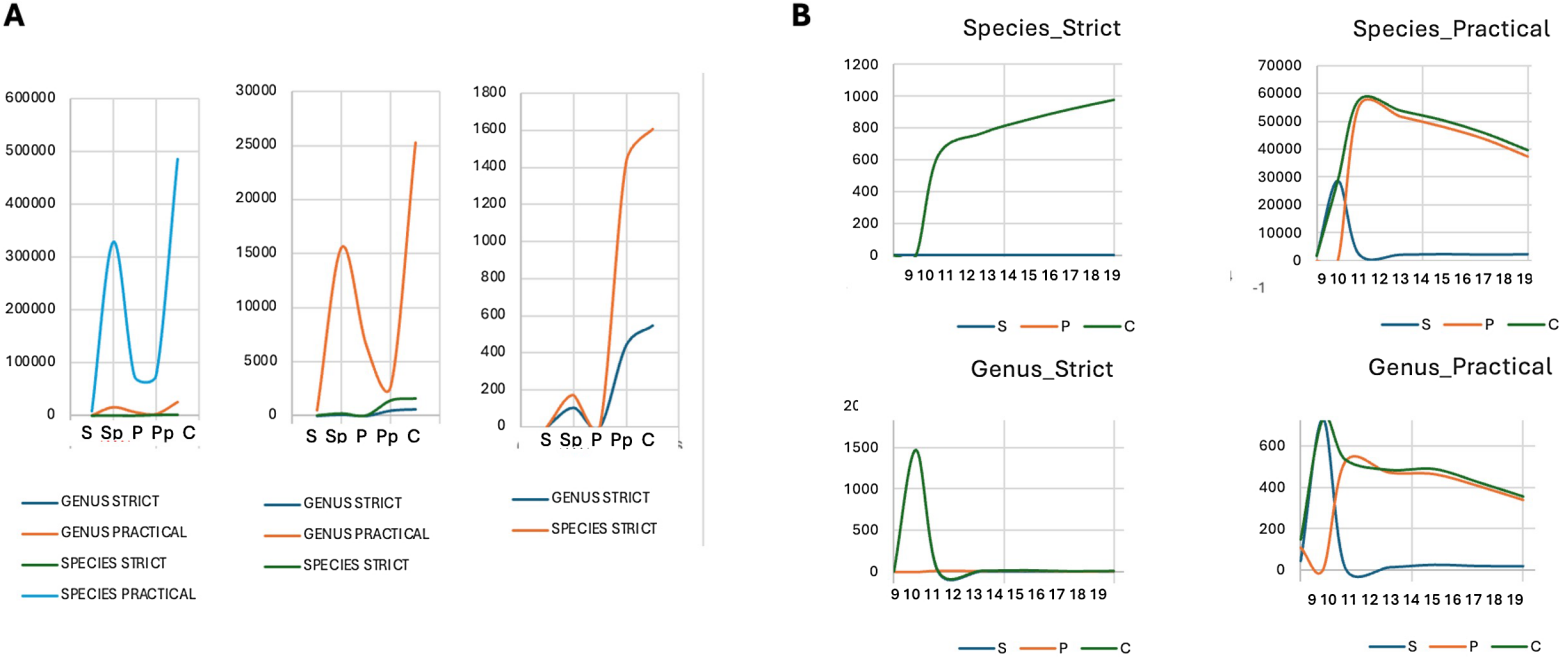
Rule counts for the two methods ALIGN and KMER. (A) Rule counts for the ALIGN approach The five character categories S, SP, P, PP and C are on the X axis and the number of rules is on the Y in all three plots. The kind of analysis is indicated by the coloured lines. (B) Rule count s for the KMER approach. The *k* values are given on the X axis and the count number is on the Y. The kinds of character S, P or C are shown with coloured lines. The raw counts can be found in Supplemental Table S2 (ALIGN) and Supplemental Table S3 (KMER).

The number and composition of diagnostic rules differed markedly across taxonomic levels and rule regimes. Under strict criteria, relatively few rules were recovered at the genus level (C = 545), compared to a substantially larger set at the species level (C = 1607). Relaxing criteria to the practical regime increased rule counts in both cases, but the effect was far more pronounced at the species level, where the total number of rules expanded more than 15‐fold (C = 174,914), compared to a ~5‐fold increase at the genus level (C = 3842).

Across all conditions, diagnostic signal for the ALIGN dataset was dominated by combinatorial rules (P and PP), which together accounted for the majority of total rules (Figure 3A). Under strict regimes, P and PP comprised ~86% of genus‐level rules and ~93% of species‐level rules, indicating that single‐site diagnostics (S and SP) contribute minimally to overall diagnosability. This pattern persisted under practical criteria, although the relative contribution of SP rules increased, particularly at the genus level (SP ≈ 39%). At the species level, however, combinatorial rules remained dominant even under relaxed thresholds (P + PP ≈ 62%).

Figure 3B (Supplemental Table S3) demonstrates a similar pattern for KMER analysis as the ALIGN sequence approach, but the number of rules is an order of magnitude lower for the practical mode versus the strict mode approach which is extremely sparse. This observation can be explained by the overly stringent strict requirement of 100% taxon agreement for k‐mers (pure characters) which is rare. Diagnostic rule performance differs markedly across taxonomic levels. While species‐level assignments achieve both high coverage and high accuracy under practical rule regimes, genus‐level assignments exhibit a strong tradeoff between coverage and resolution, reflecting distributed and overlapping diagnostic signal. In contrast to genus‐level diagnostics, which are constrained by overlapping signal, species‐level diagnostic rules achieve both high coverage and high accuracy under relaxed criteria, indicating that discriminative signal is concentrated at finer taxonomic scales (Figure 4).

**FIGURE 4:**
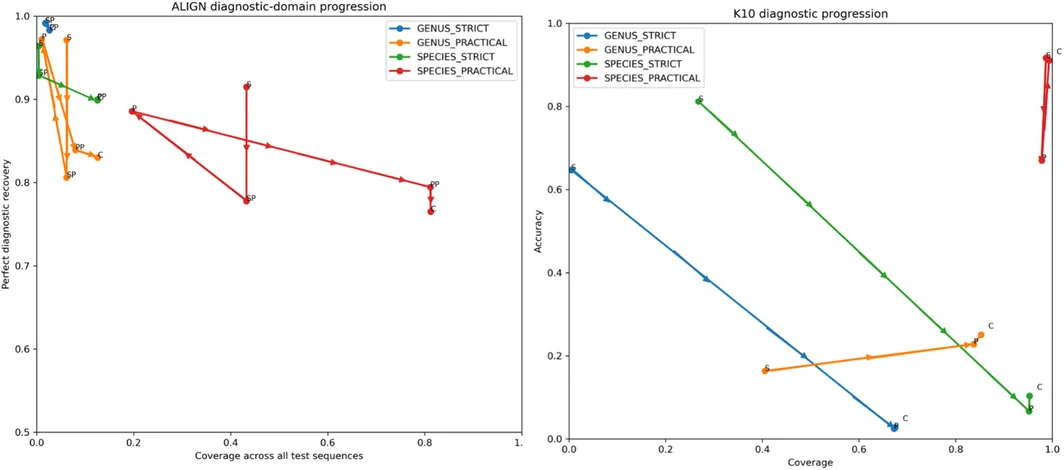
Progression of diagnostic performance across rule classes for ALIGN (left) and KMER (right) diagnostic systems under strict and practical rule‐generation regimes. Coverage (x‐axis) and accuracy (y‐axis) are shown for successive rule classes (S → SP → P → PP → C) at both genus and species levels. Left panel: ALIGN‐based diagnostics derived from positional nucleotide rules. Right panel: KMER‐based diagnostics derived from shared *k* = 10 feature space.

Notably, the dramatic expansion of rule counts under practical criteria at the species level reflects the abundance of diagnostic signal distributed across combinations of sites. In contrast, the more limited growth in genus‐level rules, coupled with a higher relative contribution of SP rules, suggests that genus‐level signal is less combinatorically structured and more diffusely distributed. These results indicate that diagnostic information is both more abundant and more strongly combinatorial at finer taxonomic scales.

Strict regimes emphasize highly specific rules with minimal tolerated conflict, whereas practical regimes allow relaxed thresholds to increase recoverability. In the ALIGN approach (ALIGN), species‐level practical diagnostics maintain high accuracy across expanding rule classes while progressively increasing coverage, whereas strict species diagnostics show a stronger tradeoff between coverage and accuracy. Genus‐level ALIGN diagnostics remain highly accurate but comparatively low coverage under strict conditions. Diagnostic progression analyses revealed fundamentally different performance dynamics between ALIGN and KMER diagnostic systems. In the ALIGN framework, expansion from singleton to increasingly combinatorial rule classes produced gradual increases in coverage while largely preserving high diagnostic accuracy, particularly under the practical regime. Species‐level practical ALIGN diagnostics remained highly accurate (> 0.8) across most rule classes while recovering progressively larger fractions of the test dataset, indicating that positional nucleotide diagnostics retain strong discriminatory signal even as rule complexity increases. Genus‐level ALIGN diagnostics showed lower recoverability under strict conditions but remained highly stable in accuracy.

In contrast, KMER diagnostics exhibit markedly different behaviour: practical species‐level diagnostics achieve near‐complete coverage with high accuracy, whereas strict KMER progression shows substantial collapse in accuracy as broader combinatorial rule classes are incorporated. KMER diagnostics exhibited a contrasting pattern. Practical species‐level KMER diagnostics achieved extremely high coverage approaching complete dataset recovery while maintaining strong accuracy, indicating abundant recoverable signal within shared k‐mer feature space. However, strict KMER progression showed pronounced degradation in accuracy as broader rule classes were incorporated, particularly at genus level, where increasing combinatorial expansion substantially increased ambiguity among competing assignments. These results suggest that alignment‐derived diagnostics retain more stable hierarchical specificity across rule classes, whereas KMER systems recover extremely broad signal but are more sensitive to combinatorial expansion and feature‐sharing among taxa. These trajectories illustrate distinct diagnostic landscapes for alignment‐based and k‐mer‐based systems and demonstrate how rule‐class expansion reshapes the balance between recoverability and precision.

Performance of practical k‐mer diagnostics varied substantially across taxonomic levels and k‐mer sizes (Figure 5). At the genus level, increasing k produced only modest improvements in diagnostic behaviour, with coverage and accuracy remaining comparatively unstable across the tested range (*k* = 9–15). Lower *k* values generated broader recoverability but poor discriminatory power, whereas larger *k* values increased specificity at the expense of consistent coverage. Overall, genus‐only k‐mer diagnostics did not exhibit a sharply defined optimum, reflecting the diffuse and overlapping nature of genus‐level signal in shared k‐mer space.

**FIGURE 5:**
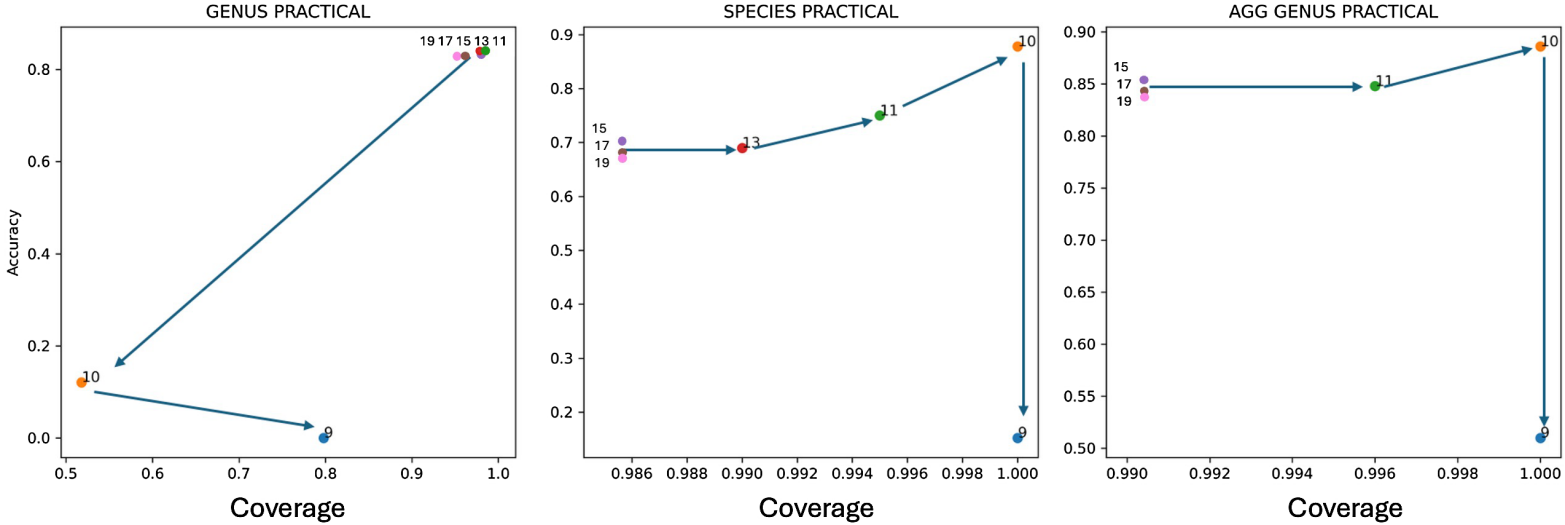
Performance of k‐mer‐based practical diagnostics across *k* (9–15). Genus‐only (left) shows limited improvement at low *k*, while species‐level rules (middle) exhibit a clear optimum at *k* = 10, with maximal coverage and accuracy. Aggregate genus assignment (right), combining genus and species‐derived also signals peaks at *k* = 10 (coverage ≈ 1.0, accuracy ≈ 0.886) and declines at higher *k*, consistent with increasing feature specificity and fragmentation of shared diagnostic signal.

In contrast, species‐level diagnostics displayed a pronounced and well‐defined optimum at *k* = 10. At this value, practical species rules simultaneously achieved near‐complete coverage and maximal accuracy, indicating an effective balance between feature specificity and recoverability. Smaller *k* values likely generate excessive feature sharing among closely related taxa, reducing assignment precision, whereas larger *k* values fragment diagnostic signal and reduce recoverability. The sharp transition observed around *k* = 10 (Figure 5) suggests that intermediate k‐mer sizes capture the most informative scale of species‐level sequence variation in the COI dataset. These results identify *k* = 10 as the optimal feature scale for downstream hybrid diagnostic modelling and deployment classification.

At *k* = 10, aggregate genus assignment (combination of KMER rules and ALIGN rules for genera) achieved both maximal coverage (1.0) and the highest observed accuracy (~0.886), outperforming all other *k* values tested. For *k* ≥ 11, aggregate accuracy declined to ~0.84–0.85 despite near‐complete coverage, indicating that increased k‐mer specificity does not translate into improved higher‐level classification when signal becomes overly partitioned. These results demonstrate that diagnostic signal is maximized at intermediate *k* (≈10), where species‐level rules provide high‐coverage, high‐accuracy assignments that effectively propagate to genus‐level classification. Both lower and higher *k* values reduce overall diagnostic performance through, respectively, signal redundancy and signal fragmentation.

### 3.2 ALIGN/KMER Hybrid Analyses

The final attempt to improve the diagnostic power of COI sequences involved combining the ALIGN and KMER rules (Figure 6). The analysis of ALIGN/KMER hybrid datasets used *k* = 10, the number of pairs examined 2 million, and the regime was practical. Hybrid progression analyses revealed strong hierarchical differences in the balance between recoverability and diagnostic precision across rule classes. Aggregate genus analyses under the strict regime exhibited comparatively low coverage at singleton and singleton‐pattern levels but maintained high assignment accuracy, indicating that conservative hybrid genus diagnostics recover only a restricted subset of confidently diagnosable sequences. Inclusion of increasingly combinatorial rule classes substantially expanded coverage but progressively reduced precision, particularly at PP and complete‐rule levels where diagnostic overlap among genera became more pronounced.

**FIGURE 6:**
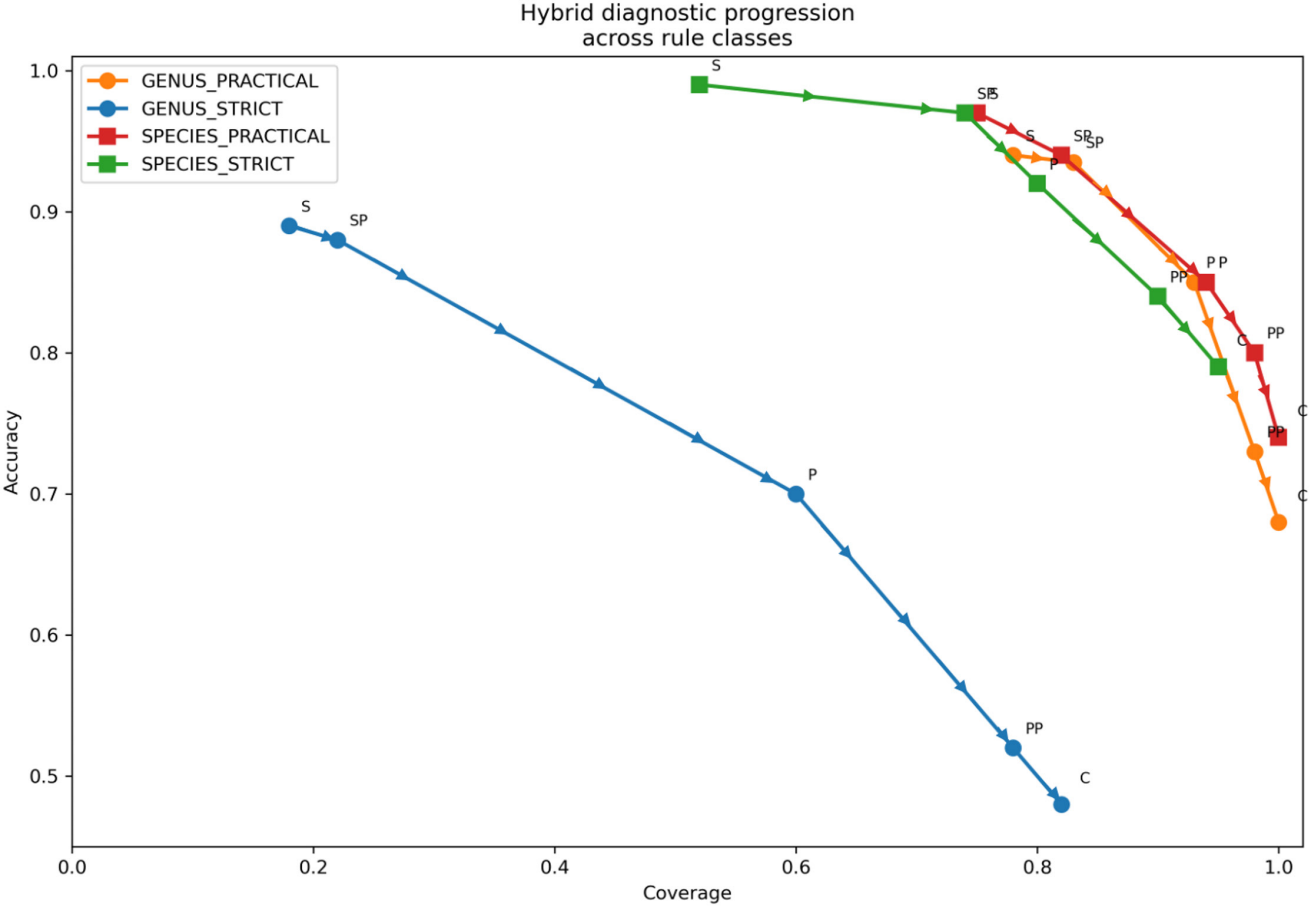
Progression of hybrid diagnostic performance across rule classes (S → SP → P → PP → C) under strict and practical regimes for aggregate genus and species‐level analyses. Coverage (x‐axis) represents the proportion of sequences receiving at least one assignment, while accuracy (y‐axis) indicates the fraction of correct assignments among resolved sequences. Hybrid diagnostics integrate aligned positional nucleotide rules and k‐mer–derived features within a unified scoring framework. Genus‐level trajectories correspond to aggregate genus analyses in which both genus‐level rules and species‐derived rules collapsed to genus identity contribute diagnostic support. Species‐level trajectories evaluate direct species assignment performance.

In contrast, practical hybrid genus diagnostics achieved substantially greater recoverability across all rule classes while retaining relatively high assignment accuracy, demonstrating that relaxed hybrid thresholds effectively recover distributed genus‐level signal. Species‐level hybrid diagnostics displayed the strongest overall performance (Figure 7). Under both strict and practical regimes, species assignments maintained high accuracy across broad coverage ranges, indicating that the combined positional and k‐mer feature space captures highly discriminative fine‐scale signal. Although increasing combinatorial complexity gradually reduced species‐level precision, the decline was substantially less severe than observed at aggregate genus level. These trajectories demonstrate that hybrid diagnostics effectively separate high‐confidence and high‐coverage assignment regimes while preserving strong discriminatory power at species scale.

**FIGURE 7:**
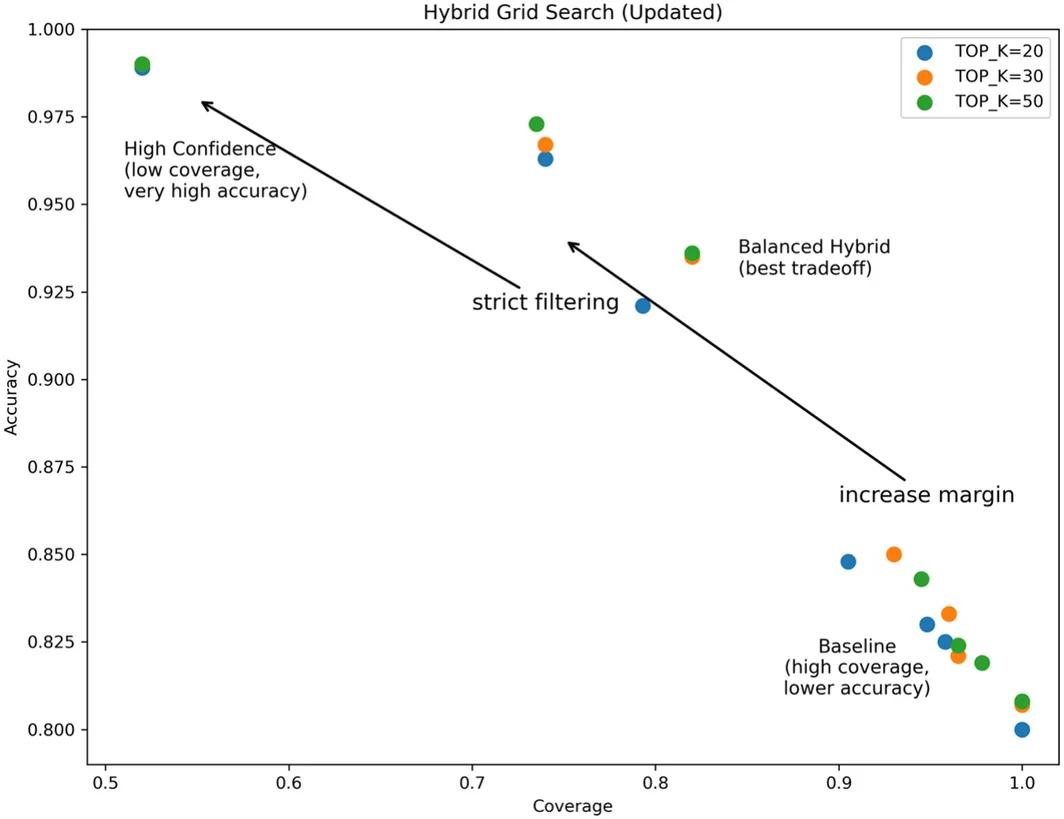
Tradeoff between coverage and accuracy in hybrid diagnostic models. Scatterplot showing classification performance across parameter combinations of the hybrid diagnostic model integrating aligned nucleotide and k‐mer features (*k* = 10). Each point represents a specific combination of feature selection (TOP_T), vote normalization and margin threshold. Three regimes are evident: (i) a *baseline regime* characterized by high coverage but lower accuracy, (ii) a *balanced regime* achieving optimal tradeoffs between coverage and accuracy and (iii) a *high‐confidence regime* with reduced coverage but near‐perfect accuracy. Arrows indicate the directional effects of increasing accuracy and coverage and stricter filtering on model performance. The balanced regime represents the optimal operating point used for downstream taxonomic assignment.

Grid‐search optimization (evaluation of all combinations of predefined parameter values) of the hybrid diagnostic framework revealed a structured tradeoff between assignment coverage and classification accuracy across parameter combinations integrating aligned nucleotide diagnostics and k‐mer features (*k* = 10). T (TOP_T) refers to the number of highest‐ranked diagnostic features retained in the hybrid voting framework during model optimization and classification. Parameter sets with permissive filtering and minimal margin constraints produced a baseline regime characterized by near‐complete sequence recovery but comparatively modest classification accuracy. Increasing filtering stringency progressively shifted model behaviour toward higher precision at the expense of reduced recoverability, demonstrating that ambiguity among competing candidate taxa can be effectively controlled through margin‐based confidence thresholds.

Across all tested values of ranked diagnostic sizes (TOP_T = 20, 30 and 50), model trajectories converged on a balanced optimum combining broad coverage with high assignment accuracy. This intermediate operating region consistently outperformed the permissive baseline while avoiding the substantial coverage collapse associated with the most stringent filtering conditions. Under maximal filtering and normalization, a high‐confidence regime emerged in which assignment accuracy approached near‐perfect levels, but coverage declined substantially, indicating that a subset of sequences contains exceptionally strong and unambiguous diagnostic signal.

The similarity of performance trajectories across TOP_T values demonstrates that the hybrid framework is robust to moderate variation in feature‐selection size and that margin‐based filtering is the dominant determinant of classification behaviour. Based on these optimization results, the balanced hybrid regime was selected as the operational configuration for downstream deployment analyses and taxonomic assignment of unknown sequences.

### 3.3 Deployment: Taxonomic Rescue

Of the > 17,000 drosophilid COI sequences in the NCBI data base, nearly 13,000 have full taxonomy. The remaining 3000–4000 are either named to genus (GENA) or named to family (Drosophilidae) or subfamily (Drosophilinae) and have neither a genus nor a species epithet (NANA) or are not in the family. The unnamed COI sequences were taken and assigned in two ways. First was an examination of the utility of the genus assignment tool. This approach uses aggregates genus rules plus species rules to assign genus. Second, the species diagnostic rules to assign species were examined. In general, the genus assignments of the aggregate method agree with the genus assignments using species rules (Figure 8).

**FIGURE 8:**
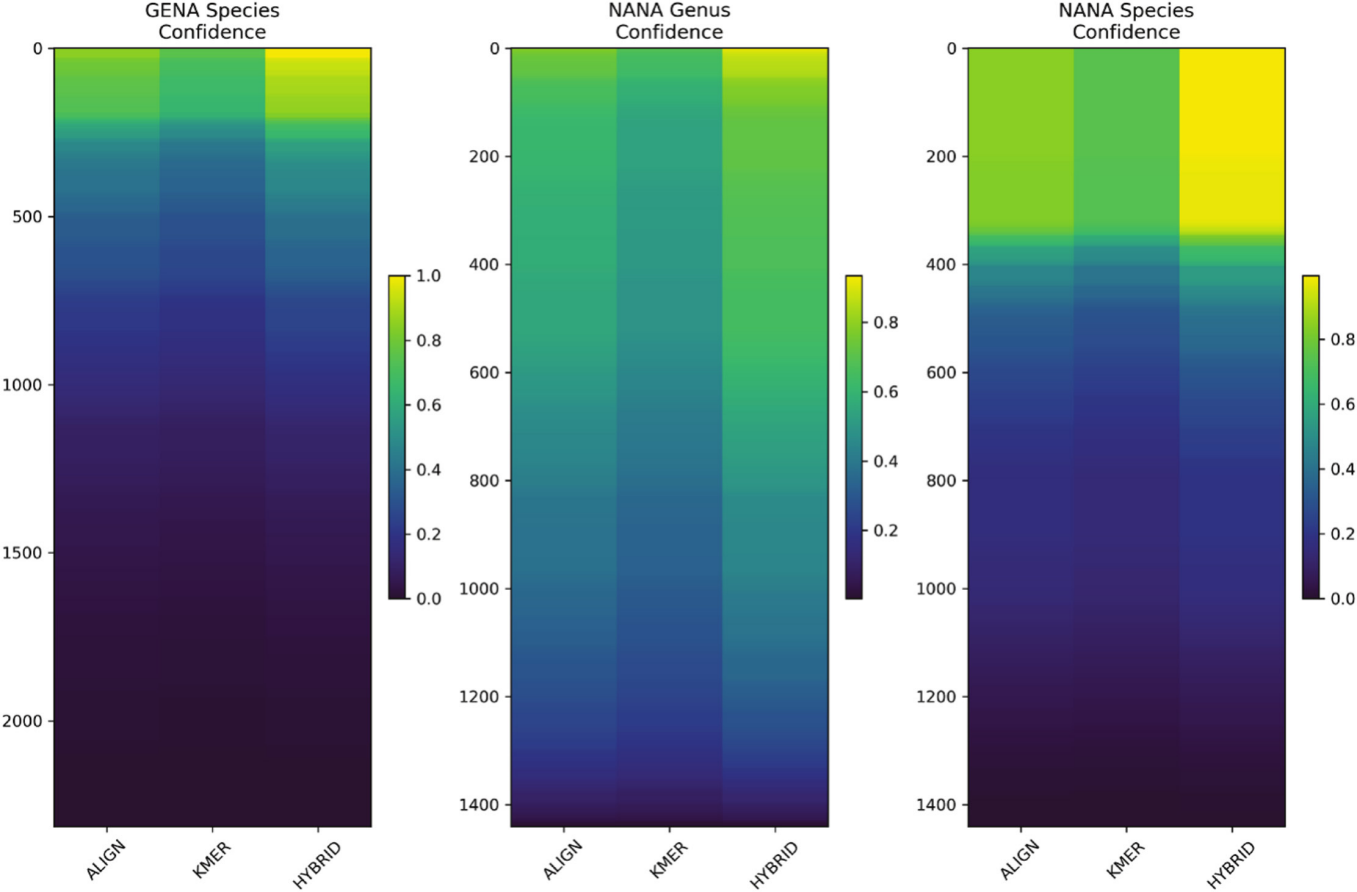
Confidence heat maps for deployment classification of unknown COI sequences using aggregate hybrid diagnostics. Rows correspond to individual sequences ranked by hybrid assignment confidence, and columns represent confidence derived from ALIGN, KMER and HYBRID diagnostic systems. Confidence values correspond to normalized separation between the highest‐ and second highest supported candidate assignments (delta score). Left: GENA (*n* = 2314) species assignments constrained by known genus identity. Middle: NANA (*n* = 14,441) genus assignments inferred de novo using aggregate genus diagnostics. Right: NANA species assignments performed following inferred genus assignment. HYBRID diagnostics generally produce the strongest and most stable confidence profiles, while ALIGN and KMER methods alone show reduced assignment separation. The heat maps reveal a pronounced hierarchical structure in diagnosability, with genus‐level assignments showing substantially stronger overall confidence than species‐level assignments.

The deployment confidence landscape (Figure 8) reveals substantial differences between genus‐ and species‐level assignment performance across the GENA and NANA datasets. In GENA, where genus identity is known and only species assignment is required, confidence is concentrated in a relatively small subset of sequences. Approximately 20% of sequences exhibit strong species‐level assignment confidence, with an additional ~20% falling into a moderate‐confidence regime, while the remaining sequences show weak separation. This pattern suggests that although species‐level diagnostic signal is present for many taxa, a large fraction of sequences occupy ambiguous regions of feature space where diagnostic support is distributed among multiple closely related species.

In contrast, genus‐level assignment in the fully unknown NANA dataset is substantially more robust. Most NANA sequences exhibit moderate‐to‐strong genus‐level support, with only a small minority (~10%) remaining weakly resolved. This result indicates that aggregate hybrid diagnostics recover genus‐level structure with high reliability even when no prior taxonomic information is available. Species‐level assignment within NANA is more challenging but still recovers substantial signal: approximately one quarter of sequences exhibit strong species‐level confidence, and roughly half can be assigned with at least moderate confidence. Together, these results demonstrate a clear hierarchical structure in diagnosability, with genus‐level signal being broadly recoverable across unknown sequences and species‐level signal concentrated in a subset of highly diagnosable taxa. In essence, nearly all COI sequences in the GENA and NANA datasets can be assigned with at least moderate confidence to a genus and nearly half of the GENA and NANA sequences can be assigned to species.

## 4 Discussion

The present analyses demonstrate that COI contains abundant recoverable diagnostic information across the family Drosophilidae, but that this information is strongly structured by taxonomic scale, sequence representation and diagnostic rule complexity. Across all diagnostic systems examined, species‐level assignments consistently exhibited greater recoverability, stronger exclusivity and lower ambiguity than genus‐level assignments. In contrast, genus‐level diagnostics were characterized by a persistent tradeoff between coverage and ambiguity, with increasingly permissive rule regimes recovering broader fractions of the dataset while simultaneously expanding candidate sets and reducing assignment precision (Rach et al. 2008; Yu et al. 2015; Zou et al. 2011; Breitling 2019). These results indicate that diagnostic signal is not distributed uniformly across hierarchical taxonomic levels. Broad taxonomic structure is widespread but overlapping, whereas species‐level signal is substantially more discriminative and recoverable for classification.

A second major result is that genomic diagnosability is fundamentally combinatorial. Across ALIGN, KMER and HYBRID systems, pairwise and higher‐order rule classes contributed a large fraction of the recoverable diagnostic structure, particularly under practical rule regimes and at the species level (Rish et al. 2002; Pazzani et al. 1994). Singleton characters alone rarely provided sufficient discriminatory power for broad classification. Expansion of pair‐space exploration revealed that large quantities of diagnostic signal remained hidden under sparse combinatorial sampling, with pairwise rule recovery increasing nonlinearly as exploration depth increased. These results suggest that taxonomic information resides primarily in distributed combinations of partially informative features rather than in isolated diagnostic characters.

Several previous studies have explored k‐mer‐based approaches in DNA barcoding and sequence classification, particularly as a means of increasing recoverable diagnostic information from short barcode fragments (Zhang et al. 2017; Bokulich 2025; Hinsinger et al. 2015; Villacrés‐Vallejo et al. 2020; Dubinkina et al. 2016). However, no previous study has explicitly integrated character‐based diagnostics with combinatorial k‐mer representations within a unified hierarchical diagnostic framework. The incorporation of paired positional diagnostics together with k‐mer‐based features greatly expands the effective diagnostic space recoverable from the relatively short COI fragment used in most barcoding applications. Rather than relying exclusively on isolated site‐specific substitutions, the present framework exploits distributed combinations of positional and compositional features that collectively capture substantial taxonomic structure.

The results of this study suggest that different sequence representations recover complementary components of taxonomic structure. Alignment‐based diagnostics exploit homologous positional characters and therefore preserve broad hierarchical organization across taxa. Although ALIGN systems frequently exhibited weaker species‐level exclusivity than KMER systems, they showed substantial stabilization following aggregation to genus level, indicating that positional signal retains higher‐order taxonomic structure even when species‐level discrimination is limited. In contrast, KMER diagnostics treat sequences as collections of compositional motifs independent of positional homology. Under this representation, species‐level discrimination became substantially stronger, with practical k‐mer systems achieving high coverage and strong assignment performance. k‐mer analyses further identified a pronounced optimum near *k* = 10. Smaller values generated abundant but weakly discriminative signal, whereas larger values fragmented diagnostic structure into increasingly sparse and taxon‐restricted features. Together, these results indicate that positional and compositional representations recover overlapping but distinct aspects of organization within the COI gene.

Hybrid diagnostic systems (combining ALIGN characters with KMER characters) demonstrated that these complementary representations can be integrated effectively within a unified framework. Initial unweighted hybrid models exhibited substantial ambiguity and unstable assignment behaviour. However, incorporation of ranked feature selection, weighted voting, normalization and margin‐based acceptance produced a clear and biologically interpretable performance structure. Hybrid systems consistently achieved a balance between coverage and assignment accuracy that could be tuned according to analytical objectives. Under stringent filtering, assignment accuracy approached near‐perfect levels, whereas more permissive parameterizations provided substantially broader recoverability. Hybrid integration therefore does not merely increase the quantity of diagnostic signal recovered but provides a flexible framework for balancing confidence and classification breadth.

The present results support and extend earlier conceptual views of DNA barcoding. Hebert et al. (2003) originally proposed that substantial discriminatory information is embedded within the relatively short COI barcode region, while subsequent character‐based approaches emphasized that diagnostic information could emerge from combinations of informative characters distributed throughout a sequence (DeSalle et al. 2005). The analyses here support these ideas and extend them by demonstrating that diagnosability is fundamentally combinatorial and recoverable across multiple representations of sequence space. This study represents the first explicit integration of positional diagnostics and combinatorial k‐mer diagnostics within a unified hierarchical diagnostic framework. These approaches should therefore be viewed as complementary rather than competing. Distance asks how similar two sequences are; phylogenetic analysis asks how they are evolutionarily related; diagnostic characters ask what sequence evidence distinguishes a taxon. Combining these perspectives provides a richer basis for taxonomic assignment than any single measure alone. This becomes particularly important as sequence databases grow, because every query may have a nearest reference even when assignment to that reference taxon is not justified.

Several caveats should be considered. The parameter values identified as optimal in the present study, including the apparent optimum at *k* ≈ 10 and the preferred feature‐selection thresholds used in hybrid analyses, are unlikely to generalize universally across all taxonomic systems. Furthermore, the present framework remains constrained by incomplete taxonomic sampling (Meyer and Paulay 2005; Smith et al. 2009; Ahrens et al. 2016). Although the NCBI drosophilid COI dataset contains approximately 17,000 accessions, only a subset possesses complete taxonomic information and sufficient sampling depth for diagnostic rule construction. Consequently, many unnamed sequences likely represent species absent from the current reference framework. Nevertheless, the diagnostic system developed here explicitly incorporates confidence estimates and permits ambiguous assignments to be rejected rather than forcing overconfident classifications. As reference sampling expands, both the breadth and reliability of recoverable diagnostic structure are expected to improve.

The taxonomy of the Drosophilidae has been the focus of more than a century of systematic investigation, resulting in one of the most comprehensive and carefully curated classifications among insect families. Rather than proposing revisions to this taxonomy, the present study builds upon this foundation by providing a reproducible framework for translating established taxonomic hypotheses into explicit DNA‐based diagnostic systems. The results reported here demonstrate that DNA‐based diagnosability is not restricted to a small number of canonical diagnostic characters. Rather, diagnosability emerges from distributed and hierarchically organized signal that can be recovered through positional, compositional and hybrid representations of sequence space. Species‐level classifications are characterized by dense and highly discriminative diagnostic structure, whereas higher taxonomic levels exhibit broader but more diffuse signal. The framework developed here therefore provides both a practical and extensible approach for recovering diagnostic information and a computational infrastructure that can facilitate future taxonomic studies, species descriptions and revisions within Drosophilidae by maintaining explicit links between nucleotide characters and taxonomic hypotheses while highlighting the fundamentally combinatorial nature of taxonomic signal within DNA barcode sequences.

## Author Contributions

R.D. conceived of the study, archived data, analyzed data, organized scripts, and wrote and edited the paper.

## Funding

The author has nothing to report.

## Ethics Statement

This study did not involve human participants or experimental procedures involving live vertebrate animals. The analyses were conducted using existing biological materials, previously generated sequence data and/or publicly available data, as described in Section 2. No animals were collected, handled or euthanized specifically for this study. Accordingly, no additional institutional animal care or human‐subjects ethical approval was required for the work reported here. Where applicable, the original sources of specimens and sequence data, including accession information, are provided in the manuscript and associated Supplementary Materials.

## Conflicts of Interest

The author declares no conflicts of interest.

## Supporting information


**Supplemental Table S1:** Truth Table for the > 17,000 drosophilid COI sequences in genbank. The accession number is given with genus then species columns.

**Supplemental Table S2:** Rule counts for the alignDNA rule set under different rule building regimes.

**Supplemental Table S3:** k‐mer sweep Counts for different *k*'s.

## Data Availability

All scripts and output files are available on DataDryad (https://doi.org/10.5061/dryad.ghx3ffc4k). Reviewer access: http://datadryad.org/share/LINK_NOT_FOR_PUBLICATION/ce‐5XiLM1rhLNSHBAJY8wGW_ZiFh8LnHyoj1s4MnXw8.
