# Supplementary material for: Species Identification in Drosophilids; the Utility of k‐mer Characters
Source: Mol Ecol. 2026 Nov 6;35(21):e70624. doi: 10.1111/mec.70624 (PMC13741199; doi:10.1111/mec.70624)
Supplement: Supplementary file 1 [file MEC-35-e70624-s002.docx]

| k | LEVEL | MODE | S | P | C |
| --- | --- | --- | --- | --- | --- |
| 9 | GENUS | STRICT | 0 | 2 | 2 |
| 9 | GENUS | PRACTICAL | 43 | 107 | 150 |
| 9 | SPECIES | STRICT | 0 | 0 | 0 |
| 9 | SPECIES | PRACTICAL | 1709 | 0 | 1709 |
| 10 | GENUS | STRICT | 1466 | 0 | 1466 |
| 10 | GENUS | PRACTICAL | 728 | 0 | 728 |
| 10 | SPECIES | STRICT | 0 | 0 | 0 |
| 10 | SPECIES | PRACTICAL | 28602 | 0 | 28602 |
| 11 | GENUS | STRICT | 0 | 13 | 13 |
| 11 | GENUS | PRACTICAL | 10 | 523 | 533 |
| 11 | SPECIES | STRICT | 0 | 623 | 623 |
| 11 | SPECIES | PRACTICAL | 2210 | 55284 | 57494 |
| 13 | GENUS | STRICT | 0 | 11 | 11 |
| 13 | GENUS | PRACTICAL | 13 | 470 | 483 |
| 13 | SPECIES | STRICT | 0 | 765 | 765 |
| 13 | SPECIES | PRACTICAL | 2036 | 51613 | 53649 |
| 15 | GENUS | STRICT | 0 | 17 | 17 |
| 15 | GENUS | PRACTICAL | 25 | 463 | 488 |
| 15 | SPECIES | STRICT | 0 | 847 | 847 |
| 15 | SPECIES | PRACTICAL | 2238 | 47986 | 50224 |
| 17 | GENUS | STRICT | 0 | 5 | 5 |
| 17 | GENUS | PRACTICAL | 19 | 405 | 424 |
| 17 | SPECIES | STRICT | 0 | 915 | 915 |
| 17 | SPECIES | PRACTICAL | 2054 | 43511 | 45565 |
| 19 | GENUS | STRICT | 0 | 9 | 9 |
| 19 | GENUS | PRACTICAL | 18 | 338 | 356 |
| 19 | SPECIES | STRICT | 0 | 974 | 974 |
| 19 | SPECIES | PRACTICAL | 2177 | 37378 | 39555 |

Supplemental Table 3. K-mer sweep Counts for different k’s.
