# Supplementary material for: Species Identification in Drosophilids; the Utility of k‐mer Characters
Source: Mol Ecol. 2026 Nov 6;35(21):e70624. doi: 10.1111/mec.70624 (PMC13741199; doi:10.1111/mec.70624)
Supplement: Supplementary file 2 [file MEC-35-e70624-s003.docx]

Supplemental Tables

| LEVEL | GENUS | GENUS | SPECIES | SPECIES |
| --- | --- | --- | --- | --- |
| MODE | STRICT | PRACTICAL | STRICT | PRACTICAL |
| S | 2 | 460 | 3 | 7916 |
| SP | 104 | 15590 | 174 | 329121 |
| P | 0 | 6485 | 0 | 72959 |
| PP | 439 | 2749 | 1430 | 76307 |
| C | 545 | 25284 | 1607 | 486303 |
|  |  |  |  |  |
| % S | 0.37 | 1.82 | 0.19 | 1.63 |
| % SP | 19.08 | 61.66 | 10.83 | 67.68 |
| % P | 0 | 25.65 | 0 | 15 |
| % PP | 80.55 | 10.87 | 88.99 | 15.69 |

Supplemental Table 2. Rule counts for the alignDNA rule set under different rule building regimes.
